# An experimental study of ultrasonic vibration and the penetration of granular material

**DOI:** 10.1098/rspa.2016.0673

**Published:** 2017-02

**Authors:** David Firstbrook, Kevin Worrall, Ryan Timoney, Francesc Suñol, Yang Gao, Patrick Harkness

**Affiliations:** 1 School of Engineering, University of Glasgow, University Avenue, Glasgow G12 8QQ, UK; 2 Department of Applied Physics, Universitat Politècnica de Catalunya-BarcelonaTech (UPC), c/ E. Terradas, 5, 08860 Castelldefels (Barcelona), Spain; 3 Surrey Space Centre, University of Surrey, Guildford, GU2 7XH, UK

**Keywords:** ultrasonic, penetration, granular, rheology

## Abstract

This work investigates the potential use of direct ultrasonic vibration as an aid to penetration of granular material. Compared with non-ultrasonic penetration, required forces have been observed to reduce by an order of magnitude. Similarly, total consumed power can be reduced by up to 27%, depending on the substrate and ultrasonic amplitude used. Tests were also carried out in high-gravity conditions, displaying a trend that suggests these benefits could be leveraged in lower gravity regimes.

## 1. Introduction

Finding signs of life, or evidence of conditions compatible with life, has long been one of the driving forces for space exploration. The subsurface of planetary bodies is an attractive environment for such a search due to shielding from the surface radiation by the ground itself. For example, the radiation at 3 m depth on Mars is no more intense than that at Earth's surface [1], and even at 1 m the radiation level is estimated to reduce to levels at which the highly radio-resistant bacteria *Deinococcus radiodurans* might survive over evolutionary time scales. In this regard, devices that are able to access this depth can have great scientific and exploratory value.

The Apollo 15–17 missions, as well as the Soviet Union's Luna 16, 20 and 24 missions, were the first to drill on another planetary body and return samples back to Earth. More recently, the Curiosity rover was designed to drill to a depth of a few centimetres on the surface of Mars [2]. However, drilling or burrowing through the ground is problematic in low-gravity situations, due to the lower overhead weight of the spacecraft. This results in a lower weight-on-bit that can be applied to the drill, often producing non-optimal drilling conditions. In addition, the total mass budget is often a severe constraint on space missions, further compounding the problem, while traditional rotary or rotary-percussive tools can require large amounts of power to operate [3].

Progress has been made with more novel instruments, utilizing high-powered ultrasonic vibration for small drilling or burrowing devices, such as the USDC developed by JPL [4], or the UPCD developed by the University of Glasgow [5]. These devices utilize a freely moving mass located between an ultrasonically tuned horn and the drill bit. When the free mass contacts the horn a large amount of momentum is transferred, causing the free mass to recoil at high speed. The free mass then impacts the drill bit, producing an impulse that can be transferred to break rocky substrates.

Innovative drills inspired by a wood-wasp's ovipositor have also been designed, using backwards-facing teeth on a split penetrator known as the dual reciprocating drill (DRD). The dual-reciprocating motion allows one half of the penetrator to generate a traction force, while the other half can drive down through the substrate [6]. Both the USDC/UPCD and the DRD mechanisms allow for significantly reduced required overhead forces for drilling. In a related system, the direct application of ultrasonic vibration has been shown to reduce required overhead penetration forces in granular material by fluidizing the surrounding substrate [7].

Systems have also been developed that rely on the whole device burrowing itself into granular material using an internal hammering system, rather than through complex drill stems and connections. These are known as ‘moles’, and require an umbilical cable attached to a ground support unit that can provide power and control to the mole. Examples of these include the PLUTO mole aboard the Beagle 2 lander [8], the MUPUS probe on the Philae lander as part of the Rosetta mission [9], and the HP3 mole on the delayed Martian InSight lander [10]. The mission requirements of the last are to penetrate 3–5 m through the Martian regolith, demonstrating that access to significant depths is achievable with low-mass devices. A summary of the key specifications of these probes is detailed in table 1.

**Table 1.** Key specifications of existing mole devices. PLUTO information taken from [8,11], MUPUS information taken from [9] and HP3 information taken from [10,12–14].

	PLUTO mole	MUPUS probe	HP3 mole
mass	0.9 kg	2.35 kg	<2 kg
length	280 mm	360 mm	350 mm
diameter	20 mm	20 mm	26.4 mm
drilling depth	1.5 m	320 mm	3–5 m
power	3 W	2.2 W	<5 W

The principal aim of the work covered in this article however is to investigate the real-world effects of ultrasonic vibration on granular rheology, and determine the general effects of the phenomenon. Modelling the quasi-fluidization of granular materials, even using spheres instead of representative grain shapes, is an enormous computational task involving literally millions of contact interactions for each ultrasonic cycle, which in itself represents less than a ten-thousandth of a second in real time. Therefore, the purpose of this work is to experimentally establish the underlying principles and how it could potentially be exploited for facilitating penetration in the style of PLUTO, MUPUS or HP3. To the best of the authors' knowledge this appears to be the first detailed examination of this effect.

## 2. Experimental apparatus

This article reports three main experiments, investigating the force, power and gravity trends with respect to ultrasonic penetration. Two experimental rigs were built to test these variables, one for the force/power tests and one for the gravity effects. As such, some components were shared and re-used between the two rigs. The common components are listed first, with the operation of the specific rigs covered subsequently.

### (a) Common components

A linear actuator provided the penetration action, either through pushing (for the force/power rig), or through pulling (for the gravity rig). In experiments where different penetration speeds were called for, voltages of 4.8 and 12.0 V were used to achieve rates of 3 and 9 mm s^−1^, respectively, where, under loading, the speed was automatically kept constant by an internal control loop that drew a higher current when required. The penetration distance was calculated via a potentiometer housed within the actuator, and penetration force was measured by a force transducer located between the linear actuator and the supporting rig.

An ultrasonic Langevin transducer (model L500 from Sonic Systems) was used to provide an ultrasonic vibration of 20 kHz, with excitation amplitude up to 10 µm. The transducer was connected to a separate signal drive system, which provided the power and allowed remote control via a computer serial interface.

**Figure 1.**
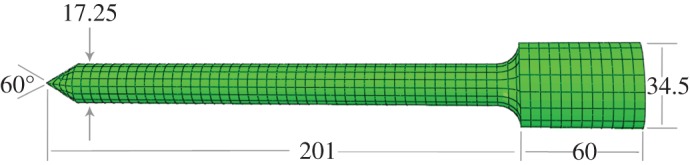
Size and shape of the ultrasonic penetrator used. Dimensions are given in millimetres. The transducer attaches at the right. (Online version in colour.)

The penetrator was manufactured from 94Ti/6Al/4 V alloy, and designed to resonate in the L2 mode at 20 kHz. The shape of the penetrator, shown in figure 1, was designed to amplify the ultrasonic amplitude provided by the transducer. The penetrator used in this article was tested using experimental modal analysis, and was found to have an amplification ratio, or gain, of 3.5, meaning 1 µm of vibration at the base provided 3.5 µm of vibration at the tip.

### (b) Force and power rig

**Figure 2.**
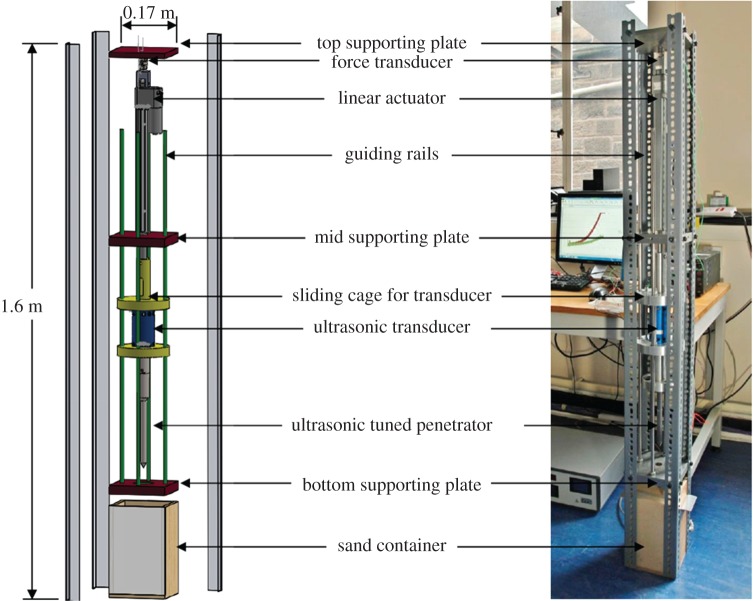
The force/power rig. (Online version in colour.)

This rig, shown in figure 2, was used for two sets of experiments with very minor modifications for each: a penetration force experiment conducted at the Surrey Space Centre, and a power optimization experiment conducted at the University of Glasgow. The force transducer, the actuator and the penetrator are arranged in a linear fashion throughout.

### (c) Gravity rig

Since Earth possesses the largest surface gravity of any rocky body in the solar system, any application of extra-terrestrial drilling or penetration will take place in lower gravity. A common low-cost method to mimic the effects of low gravity in the laboratory is to use counter-balances to reduce the effective weight of the experimental equipment, which has proven to be effective in drilling small distances though rock. However, this method is less appropriate for penetration through granular materials, since the movement and flow of sand is very dependent on the gravitational acceleration itself.

For more representative low-gravity experiments, there are three main options: drop towers, parabolic flights and in-orbit experiments. Drop towers can offer several seconds of microgravity, and parabolic flights can improve on this time scale by providing up to 20 s of Martian gravity conditions [15]. This is closer to the time required for a single penetration run, but only a few experiments can be done in a single day. Orbital experiments are even more challenging.

A more effective first approach is to conduct experiments at higher gravity, in a centrifuge, and extrapolate results downwards. This allows an extended experimental programme (in fact, around 400 separate runs were carried out to establish repeatable trends) which, for the purposes of this work, was carried out in the large diameter centrifuge (LDC) at the European Space Research and Technology Centre (ESTEC). Extrapolated results are seldom as accurate as direct measurements, but with such a large amount of experimental runs, these results will serve as a very effective indicator on which to potentially base future testing.

**Figure 3.**
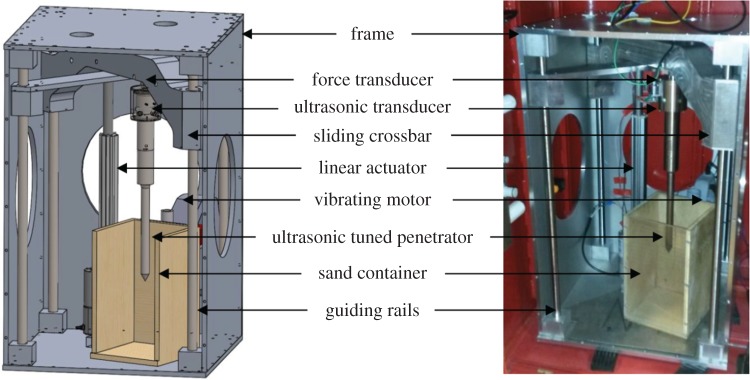
The gravity rig. (Online version in colour.)

The experimental apparatus had to be modified to fit within the LDC gondolas, shown in figure 3, ruling out the previous force/power rig. To solve this issue, the design was essentially ‘folded’, with the actuator pulling on a cross-bar which was in turn connected to the penetrator.

This method has the potential to produce a lot of torque due to the off-axis forces from the actuator and penetrator, so a thick cross-bar was manufactured to be able to withstand these forces. The force transducer was, once again, placed directly above the penetrator.

## 3. Experimental characterization and calibration

Any drilling or penetration test requires accurate characterization of the substrate used and the manner in which it was prepared, to allow for reproducible results. This is perhaps not as straightforward for granular materials compared with solids due to the quasi-fluidic nature of sand, and so different considerations are warranted. The following section will explain the various measurements and method of sample production of the sands used, as well as a description of the experimental procedure.

### (a) Sand measurements

#### (i) Sand particle distributions

**Figure 4.**
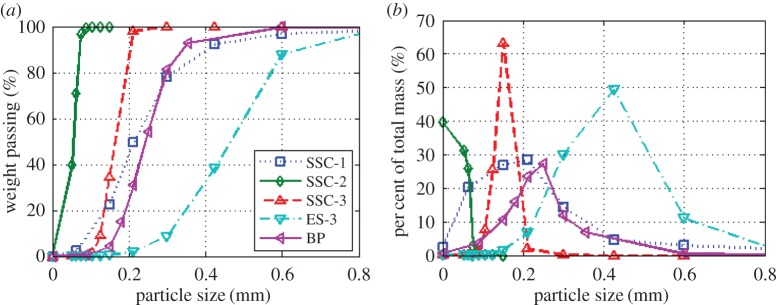
Sand particle size distributions: (*a*) by cumulative percentage weight passing, (*b*) by percentage of total mass. (Online version in colour.)

Initial tests used a total of five different regolith simulants. Four of these were published simulants SSC-1, SSC-2, SSC-3 and ES-3, with an additional block paving sand, referred to as ‘BP’ in this article [7,16–18]. The simulants were run through a set of progressively finer sieves, and the resultant sand mass of each division was measured to calculate the distribution of particle sizes within the samples. Two common methods of particle size distributions, cumulative percentage weight passing, and percentage of total mass, are shown in figure 4*a*,*b*, respectively. These graphs show the broad range of particle sizes covered by the five regolith simulants.

#### (ii) Sand particle density

The particle density of a granular material is the density of an individual particle. As opposed to bulk density, which incorporates the void space within a volume of sand, the particle density is an inherent property and does not vary with compaction. Most of the particle densities in this study were near the density of quartz, 2.65 g cc^−1^, although the absolute value varied depending on the percentage of other minerals within the sand in question (SSC-2, a garnet sand, was the only sand not based on quartz and thus had a notably higher particle density).

To determine this density in each case, a known mass of material must be submerged in a measured volume of water. The mass of the material can then be divided by the volume of fluid displaced, thereby giving the density of the material. This was the ASTM standard method [19] as used on the lunar regolith samples from the Apollo missions to characterize the particle density of lunar regolith [20]. The measured particle densities of sands BP and SSC-3, as well as the literature values for the other sands [21], are shown in table 2, along with the standard deviation (s.d.).

**Table 2.** Particle density of the sands used. Literature values from [21] are indicated by an asterisk.

sand	particle density (g ^−1^ cc)	s.d.
SSC-1	2.394*	0.057
SSC-2	3.154*	0.264
SSC-3	2.630	0.050
ES-3	2.599*	0.057
BP	2.629	0.013

#### (iii) Minimum sand bulk densities

The method used in this work to calculate the minimum bulk density follows the American Society for Testing and Materials (ASTM) method C, which is recommended for granular materials with 100% of all particles under 9.5 mm, and less than 10% above 2 mm [22]. This method consists of filling a cylinder with a known mass of sand, tipping the container over to loosen the sample, and reading off the final volume once a consistent reading is achieved. The average of these three readings is given in table 3.

**Table 3.** Minimum bulk density of sands used. Literature values from [21] are indicated by an asterisk.

sand	minimum density (g ^−1^ cc)	s.d.
SSC-1	1.384*	0.0133
SSC-2	1.949*	0.0129
SSC-3	1.381	0.0019
ES-3	1.498*	0.0187
BP	1.405	0.0034

#### (iv) Maximum sand bulk densities

Many experimental methods exist for establishing the maximum bulk density of a granular material. For this work, we follow the same technique used for the sands SSC-1, SSC-2 and ES-3, which can be found in [21]. The maximum bulk densities were calculated from the particle density figures and a 25% void ratio. The results are shown in table 4.

**Table 4.** Estimated values of maximum bulk density using a void ratio of 25%. Literature values from [21] are indicated by an asterisk.

sand	maximum density (g ^−1^ cc)	s.d.
SSC-1	1.795*	0.0428
SSC-2	2.366*	0.198
SSC-3	1.972	0.0378
ES-3	1.914*	0.0428
BP	1.971	0.00947

#### (v) Relative densities

One of the largest contributors to the resistive force encountered during penetration is the relative density of the granular material. A given sample of sand can either be extremely loose (0% relative density, or minimum theoretical bulk density), or highly compacted (100% relative density, or maximum theoretical bulk density). This level of compaction depends on the preparation method, as will be discussed in §3b. Owing to the specific ASTM method selected, however, it is sometimes possible to have negative relative densities if the sand loosening process ultimately used in practice is particularly effective. The bulk and relative densities of the five sands used are given in table 5. It is interesting to note that while SSC-1, SSC-3, ES-3 and BP are all quartz based and therefore have similar particle densities, they all vary in their compact bulk density values. Sands with high amounts of fine particles, such as SSC-1, tend to exhibit higher relative densities, as the smaller particles will fill in the voids left by the larger grains. Additionally, SSC-2 is garnet based and a very small particle size, leading to higher values of bulk density.

**Table 5.** Bulk density and corresponding relative density of the five sands used in the force experiments, along with the standard deviations for bulk density taken over eight measurements. Different scales were used for SSC-3 and ES-3, with all measurements falling within the 50 g weight sensitivity, thus giving a s.d. value of zero. Owing to the method of establishing the lowest and highest achievable densities, negative relative densities are possible.

	bulk density (g ^−1^ cc)				relative density (%)	
sand	loose	s.d.	compact	s.d.	loose	compact
SSC-1	1.408	0.0023	1.709	0.0047	7.4	83.0
SSC-2	1.882	0.0061	2.229	0.0043	−20.3	71.3
SSC-3	1.384	0	1.595	0	0.7	44.7
ES-3	1.470	0	1.671	0	−8.8	47.7
BP	1.432	0.0013	1.626	0.0013	6.5	47.4

### (b) Sand preparation methods

The method by which a sample of sand is prepared affects its final bulk density, and by extension the final penetration force [23]. For these experiments, loose samples of sands were prepared by suspending a hopper of sand and allowing it to fall into the final container. It has been shown that fall heights in excess of 50 cm allow sand to reach terminal velocity, resulting in a final homogenous distribution [24]. These preparations used a fall height of 1 m to ensure that the 50 cm limit remained satisfied even as the container filled with sand.

To produce compact samples of sand, a vibrating motor was attached to the sand container. It was turned on before allowing the sand to fall, and turned off as soon as it was full. The apparatus was not touched during filling, resulting in very consistent final density readings, typically within 0.5%.

**Figure 5.**
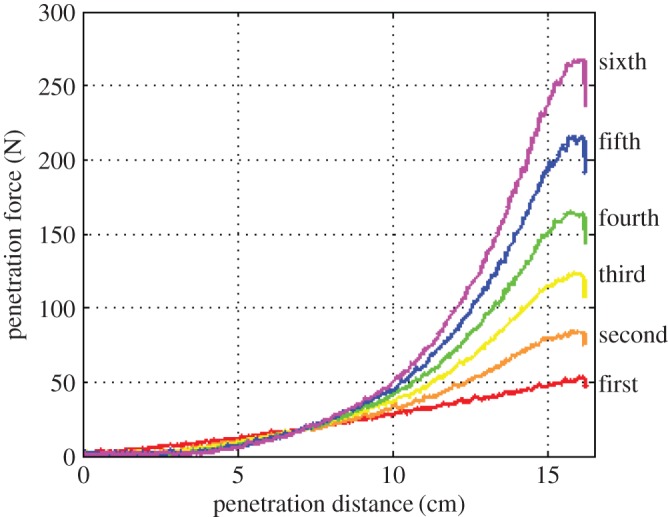
Effects of consecutive penetrations into low density ES-3 without sample reset. Each test densifies the deep sand, causing subsequent penetrations to experience a larger force. (Online version in colour.)

A new sample of sand is required for each test, since the packing structure of the sand is altered by the previous run. Figure 5 shows the effect of consecutive penetrations into sand without any resetting in between, where the peak penetration force can be seen to increase by roughly 50 N each time. This effect is due to settling effects within the sample, with each penetration run further compacting the material [25]. The sharp drop in force at the end of each run was seen across all tests, and is due to relaxation of the sand particles once the penetrator comes to a stop.

This method of sand preparation was not feasible with the gravity rig, as it was locked within a centrifuge gondola and therefore difficult to access. Instead, a smaller amount of sand was placed in the container, and the container vibrated for 1 min to reset the sand *in situ*. Penetration forces were consistent between vibrations, indicating that the sand had been completely reset in this time.

Ideally, any penetration experiment will be into an infinite sized container of sand, eliminating any boundary effects with the container wall. The container-to-penetrator diameter ratio has been shown to affect penetration resistances, with low ratios showing comparatively higher resistance than higher ratios [26]. This is heavily dependent on relative densities, however, with loose samples of sand displaying very little variation in encountered resistance. The container size of 14 × 14 × 25 cm was a compromise between allowing a sufficiently large diameter ratio (between 8.1 on the edge and 11.5 on the diagonal), while also being small enough to fit within the experimental rigs. Additionally, the power required to excite the entire sample has to be kept within the operational limits of the vibrating motor and, while a larger container would reduce boundary effects, all tests used the same container to ensure that any of these effects are consistent across all tests.

### (c) Experimental procedure

For the force/power experiment, a container of sand was first prepared using the free-fall method described in §3b. The container was then placed within the rig, and a distance reading taken for the zero point. The voltage to the actuator was set to give either a slow or fast penetration rate, at 3 mm s^−1^ or 9 mm s^−1^, respectively. The penetrator was then raised to the starting position, and ultrasonic vibration initialized. Running a custom Matlab script allowed data to begin recording, before switching the actuator on to begin penetration until the maximum depth was reached. Finally, the ultrasonic signal was cut, the penetrator was raised out of the container, and the container was replaced within the sand preparation area ready to begin a new test.

Owing to the different rig designs, the experimental procedure for the force/power experiment and the gravity experiment also had to be altered. The sand container was filled with a set amount of sand, and loosely secured within the testing rig to allow a small amount of vibration. The entire rig was then placed within the centrifuge, with the team re-locating to the control room for safety. A different Matlab script was used which automated the entire experimental process. First, the penetrator was raised to its starting position, and the container vibrated for 1 min to reset the sand. The centrifuge was then spun to a specific *g* level, and the ultrasonic vibration set to a specific amplitude.

Once the centrifuge was giving a stable *g* level, data acquisition was started, and the actuator turned on to begin penetration. Once the maximum depth was reached, data acquisition and the ultrasonic vibration were stopped, and the centrifuge was spun down to 1*g* (stationary). Once stationary, the penetrator was able to be remotely raised to the starting position again, ready for further experiments. Raising the penetrator and vibration of the container were done in 1*g* and not at higher levels of gravity in order to reduce stress on the components, as well as to reset the sand in a consistent environment.

## 4. Results

Three main phenomena of ultrasonic penetration were investigated, namely force reduction, power reduction and gravitational effects. This section presents the results obtained using the two rigs described in §2.

### (a) Force reduction

**Figure 6.**
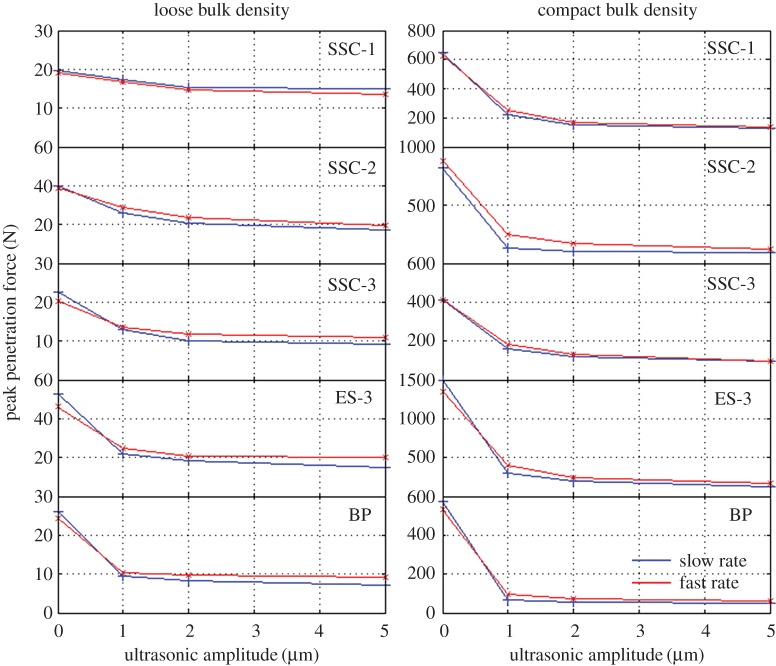
Maximum encountered penetration force as a function of ultrasonic amplitude used [7]. (Online version in colour.)

Using the force/power rig, initial tests investigating the effects of ultrasonic amplitude, penetration speed, sand choice and relative density were conducted [7], as presented in figure 6. These tests showed that application of ultrasonic vibration has a large impact on the peak penetration force, where the greatest reduction occurred at the lowest vibration amplitude, 1 µm, with diminishing returns at higher amplitudes.

Two rates were used in these first tests, slow and fast, at 3 mm s^−1^ and 9 mm s^−1^, respectively. Slight differences in the resultant peak force were noted, with the slower rate resulting in a lower force for ultrasonic penetration than the faster rate. Conversely, for non-ultrasonic penetration, the opposite was true.

Five different sands were also investigated, showing some variations in maximum penetration force. The sand ES-3, in particular, provided a peak ultrasonic force almost double that of the other sands.

Finally, loose and compact relative density samples prepared in accordance with §3b were tested, with the high-density runs in general showing a larger decrease in penetration force upon the application of ultrasonics. This is likely due to the fact that low-density samples of sand are not a stable configuration of particles, collapsing with very slight disturbances and allowing low-force penetration regardless of any additional measures.

#### (i) Parameter scoping

Given these initial findings, further tests in both the force/power rig and the gravity rig considered the 0–2 µm amplitude range only, to allow for higher resolution in the region of greatest change. Subsequent tests also used just a single penetration rate, with the faster rate of 9 mm s^−1^ being chosen as this corresponds to the linear actuator's optimum operating input voltage of 12 V, while the SSC-3 and BP simulants were considered to be broadly representative of the overall substrate characteristics. Finally, further tests focused on high-density samples, as these show the greatest range of penetration forces.

### (b) Total power consumption of ultrasonic penetration

**Figure 7.**
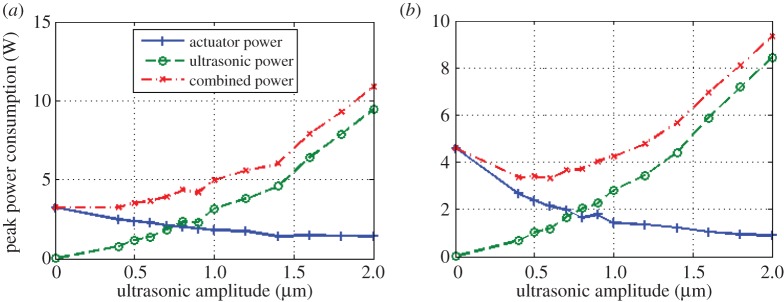
Peak power consumption during penetration through regoliths: (*a*) SSC-3 and (*b*) BP. (Online version in colour.)

In addition to reducing maximum penetration forces, ultrasonics also has the potential to reduce the peak power consumption of penetration [27]. The power consumption, depicted in figure 7*a*,*b*, corresponds to the peak power encountered at the deepest point of penetration, and the trade between low ultrasonic power and the higher actuator power needed to overcome greater resistance (and vice versa) is immediately apparent.

In SSC-3, the benefits of ultrasonics are solely limited to force reduction, but in BP the force reduction leads to such marked reductions in actuator power that the overall power requirements are themselves reduced.

Further investigation of the results indicated that similar trends exist for total energy consumption.

### (c) Maximum penetration force of ultrasonic penetration under various gravity levels

**Figure 8.**
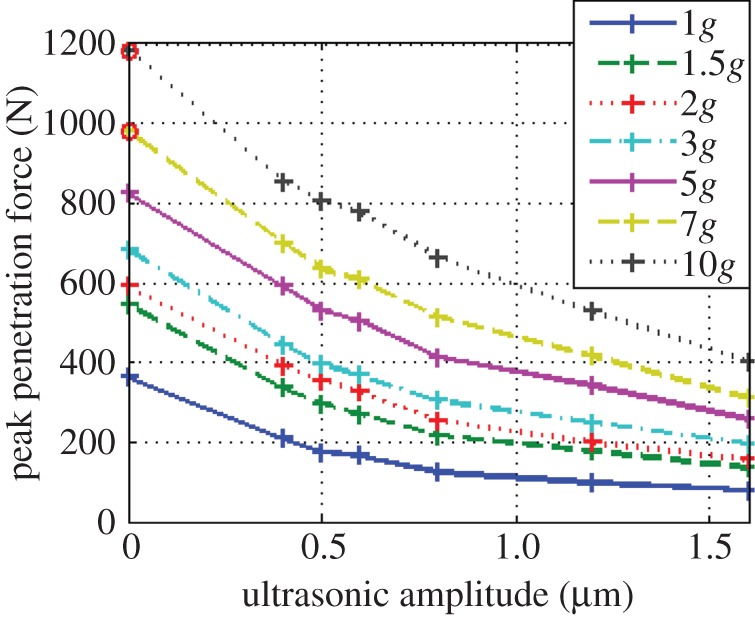
Peak penetration forces in various levels of gravity. The circled data points are estimated values. (Online version in colour.)

The experiments at the LDC were focused on mild levels of hypergravity, in order to better extrapolate trends for below 1*g* [28]. To maximize experimental time, only BP was used for these experiments, and the ultrasonic amplitude range was further decreased to 0–1.6 µm. The maximum penetration forces encountered in each experiment is shown in figure 8, in a similar manner with respect to the graphs shown in figure 6. The force-reducing property of ultrasonic vibration is still visible at higher gravities, with the lowest forces coinciding with the highest levels of vibration. The non-ultrasonic runs at 7*g* and 10*g* were not carried out due to excessive anticipated forces, but estimated placeholder points at 0 µm for 7*g* and 10*g* in figure 8 are provided and circled to help visualize the trends. These two values were calculated by extrapolating the trend seen in the force reduction between 0 and 0.4 µm at the other gravity levels, and adding it to the forces at 0.4 µm for 7*g* and 10*g*.

**Figure 9.**
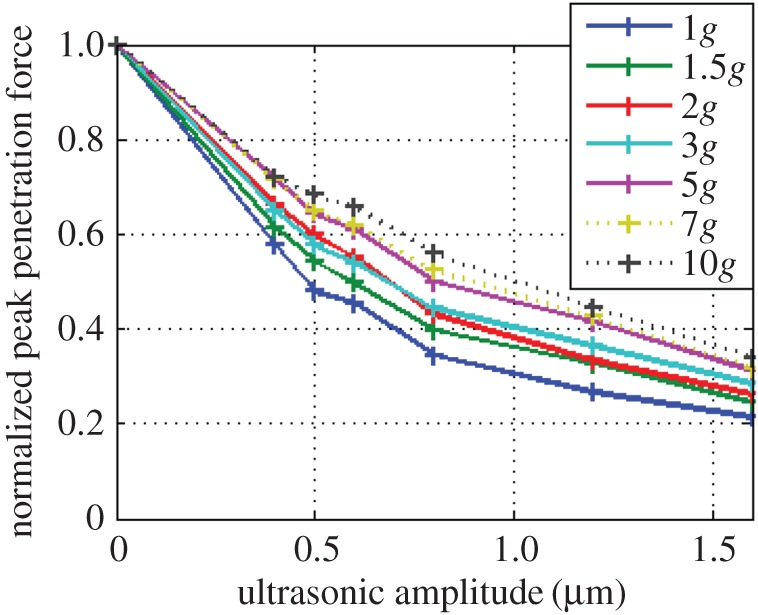
Peak penetration forces normalized to the non-ultrasonic force. The data plots for 7*g* and 10*g* are dotted to illustrate the 0 µm values (on which the normalisation is based) are estimates. (Online version in colour.)

By normalizing each penetration force with respect to the force encountered at 0 µm to create a dimensionless ratio, it is possible to directly compare the effectiveness of ultrasonic vibration on penetration forces, as seen in figure 9. The trends for 7*g* and 10*g* are dotted to illustrate that these are based on the estimated 0 µm placeholder values only.

## 5. Discussion

The results in §4 represent of a very large number of experiments and warrant an examination and discussion of the implications, as described in the following section.

### (a) Effects of ultrasonic vibration on peak penetration force

These tests were conducted up to 5 µm in order to obtain a broad range of amplitudes to investigate the force-reduction phenomenon. Owing to the ultrasonic vibration, sand in contact with the penetrator will tend to move, resulting in a ‘fluidization’ of the sand immediately surrounding the probe. This effect also occurs at lower frequencies, and has successfully been used to reduce the weight needed to insert piles for building foundations [29].

As can be seen in figure 6, the application of ultrasonic vibration decreases the required penetration force across all instances. Diminishing returns are seen above the lowest amplitude of vibration used (1 µm), resulting in further tests concentrating at the lower amplitudes. Lower amplitudes also have the advantage that less power is consumed by the ultrasonic transducer.

Slight differences can be seen in the peak resistance forces of the slow and fast penetration experiments. There appears to be a general trend that the slower penetrations at 0 µm result in a larger force, while the opposite is true when ultrasonic vibration is used (as is evident by the slow and fast plots crossing). This could be related to the duration of time that the sand has to interact with the ultrasonic penetrator, with a longer contact time resulting in a higher degree of vibration-induced fluidization.

### (b) Effects of ultrasonic vibration on total power consumption

Since ultrasonic vibration reduces penetration force, the actuator power will also decrease. However, the ultrasonic transducer requires electrical power to provide the vibration, increasing with larger amplitudes. Total power consumption therefore becomes a balancing act between the decreasing actuator power and the increasing ultrasonic power.

The acoustic power dissipated by the ultrasonic transducer is measured by the ultrasonic power supply. The power required for penetration can be calculated from simple work equations, with the instantaneous penetration power calculated to be the product of penetration force and rate.

Both of these peak powers levels, as well as the sum of the two, are plotted in figure 7*a*,*b*. The experimental runs in BP show a minimum in combined power draw at 0.5 µm, implying that this level of vibration is the optimum ultrasonic amplitude for the most efficient penetration. At this level of excitation amplitude, the total power consumption is reduced by 21.3% compared with non-ultrasonic penetration. There is no such clear optimum excitation amplitude with SSC-3 regolith, however, suggesting that total power reductions for ultrasonic penetration can heavily depend on the specific substrate.

It is prudent to take a note that although the total power draw in BP is lower, the actual optimum amplitude will depend on each specific task. If a spacecraft has an excess of power available but is extremely low-mass (e.g. a tethered mole), then the highest available amplitude would be the correct choice for this instance as it would reduce the force requirements by the largest amount.

The power measurement experiments were repeated in higher gravities using the gravity rig, where total power consumption was shown to reduce consistently at higher gravities, with a maximum value of 27% reduction obtained [28].

### (c) Ultrasonic vibration in higher gravities

The force-reduction properties of ultrasonic vibration remain at higher gravities, indicating that it is not an effect unique to Earth's gravity. As shown in figure 8, the penetration forces across all ultrasonic amplitudes increase at higher gravity. We are confident that this is not due to higher compaction of the sand at higher *g* levels, since the sand is reset in 1*g*, and the level of the surface was not seen to decrease during high *g*-loading (as viewed through an in-gondola camera), suggesting that the bulk density remained constant.

Instead, this is a feature of the *unit-weight* of sand increasing, which is found in some theoretical models for force on foundation piles and varies with gravity [30]. This suggests that required penetration forces will be lower on other planetary bodies. However, the weight of the lander or rover (and thus the maximum overhead penetration force that can be exerted) will also decrease in lower gravity, potentially negating this effect.

Figure 9 presents the percentage by which an ultrasonically assisted penetration force may be reduced, compared with its non-ultrasonic counterpart. For example, taking an amplitude of 1 µm in Earth's gravity, the force can be reduced by 70%. Comparatively, using the same vibration amplitude in 10 times Earth's gravity yields a force reduction of just 50%.

**Figure 10.**
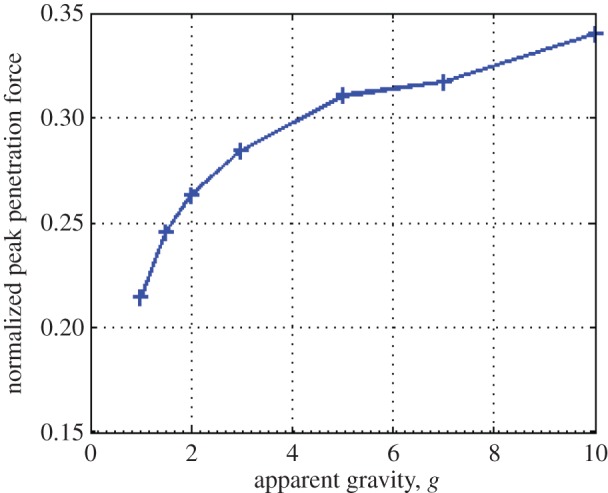
Normalized peak penetration forces at 1.6 µm with respect to gravity. Lower gravities show proportionally less force required than higher gravities, using the same vibration amplitude. (Online version in colour.)

Another way to look at this data is presented in figure 10, which takes the normalized peak penetration forces encountered at the maximum ultrasonic vibration amplitude of 1.6 µm, and plots them against their respective gravity level. Extending this trend, it is possible to infer that granular penetration in lower gravity environments will experience an even greater percentage decrease in force for a given ultrasonic amplitude.

From an applications standpoint, reducing the weight on bit requirement in low gravity could allow small landers easier access to greater depths, potentially increasing the amount of science they could perform. The authors recognize that tests conducted in low gravity will yield a greater insight into the force-reduction properties of ultrasonic penetration, but extrapolating results from higher gravity the tests described in this article show a promising start for further investigation.

## 6. Applications

The device used in these experiments was never envisioned to be a final tool for use in space applications. Instead, it is intended to examine the effects of ultrasonic vibration on granular material, and highlight it as a *technique* that can be applied to other drilling or penetrating systems in order to benefit the operation as a whole.

For example, ultrasonics could potentially be used in anchoring systems. Anchors are an attractive option for small landers, providing an additional reaction to increase the effective overhead force. However, this means that an anchor is normally extremely difficult to remove after its intended use, potentially even needing to be cut away and sacrificed so that the lander or rover can again mobilize.

**Figure 11.**
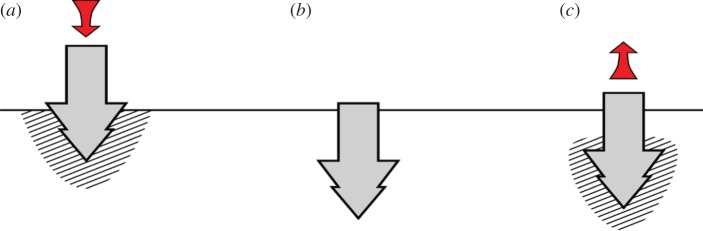
Illustrated example of using a controllable anchor. (*a*) The penetrator is inserted with ultrasonics on, creating a region of fluidized sand and reducing the required force for penetration. (*b*) The ultrasonics is switched off, returning the sand to its motionless state, and allowing the anchor to function normally. (*c*) When anchoring is no longer needed, the ultrasonics is switched back on, fluidizing the sand and reducing the required force for withdrawal. (Online version in colour.)

By utilizing the fluidized nature of ultrasonically excited sand, however, it could be possible to produce a controllable anchor. Driving an anchor through the ground while it is vibrating ultrasonically would allow it to reach greater depths using the same overhead weight, allowing a more secure placement. Turning the ultrasonics off would allow the anchor to function as normal. Additionally, since the ultrasonic vibration causes the surrounding sand to fluidize, the sand particles could fill in any voids left by the movement of the penetrator, giving it a higher degree of traction once the vibration is ultimately switched off. For recovery of the anchor, activating ultrasonics could again fluidize the sand around it, reducing the retrieval force needed. A visual representation of this process is shown in figure 11.

This is just one example of the potential impact that ultrasonics could have in the field of low-mass/low-gravity exploration of extraterrestrial bodies. It is hoped that this article might serve as an initial seed to open up further research on this topic, encouraging additional discussion and collaboration.

## 7. Conclusion

The work carried out in this article has concentrated on establishing the effects and phenomena of direct ultrasonic vibration on granular material. It has been shown that ultrasonic vibration is able to significantly reduce the required overhead force for penetration through granular material by over an order of magnitude, depending on the level of vibration used and the target material. Additionally, by using an optimum level of ultrasonic vibration, the total power consumption can be reduced by just over 20%. These effects can occur together and are predicted to improve still further in low-gravity environments.

## Supplementary Material

Raw data of figures for paper "An Experimental Study of Ultrasonic Vibration and the Penetration of Granular Material"

## Data accessibility

The data supporting this publication is available in the electronic supplementary material.
